# Neuro-nanotechnology: diagnostic and therapeutic nano-based strategies in applied neuroscience

**DOI:** 10.1186/s12938-022-01062-y

**Published:** 2023-01-02

**Authors:** Leili Shabani, Milad Abbasi, Zeynab Azarnew, Ali Mohammad Amani, Ahmad Vaez

**Affiliations:** 1 Department of Emergency Medicine, School of Medicine, Namazi Teaching Hospital, Shiraz University of Medical Sciences, Shiraz, Iran; 2 Department of Medical Nanotechnology, School of Advanced Medical Sciences and Technologies, Shiraz University of Medical Sciences, Shiraz, Iran; 3 Department of Tissue Engineering and Applied Cell Sciences, School of Advanced Medical Sciences and Technologies, Shiraz University of Medical Sciences, Shiraz, Iran

**Keywords:** Nanotechnology, Nanoparticles, Nanomaterials, Neuroscience

## Abstract

Artificial, de-novo manufactured materials (with controlled nano-sized characteristics) have been progressively used by neuroscientists during the last several decades. The introduction of novel implantable bioelectronics interfaces that are better suited to their biological targets is one example of an innovation that has emerged as a result of advanced nanostructures and implantable bioelectronics interfaces, which has increased the potential of prostheses and neural interfaces. The unique physical–chemical properties of nanoparticles have also facilitated the development of novel imaging instruments for advanced laboratory systems, as well as intelligently manufactured scaffolds and microelectrodes and other technologies designed to increase our understanding of neural tissue processes. The incorporation of nanotechnology into physiology and cell biology enables the tailoring of molecular interactions. This involves unique interactions with neurons and glial cells in neuroscience. Technology solutions intended to effectively interact with neuronal cells, improved molecular-based diagnostic techniques, biomaterials and hybridized compounds utilized for neural regeneration, neuroprotection, and targeted delivery of medicines as well as small chemicals across the blood–brain barrier are all purposes of the present article.

## Background

The fundamental components of the nervous system are neurons. When several neurons are fired together, their coordinated firing activity forms functional circuits in the brain [1]. Neurological injuries and illnesses such as cancer, traumatic brain injury, and neurodegenerative disorders may be caused by the nervous system's susceptibility [2]. Strategies now in use, which include radiation therapy, chemotherapy, and surgery, do not meet expectations in terms of lowering death rates. Patients who survive often have an unsatisfactory quality of life that follows. Because there is no efficient and optimum treatment, the challenges associated with the nervous system are mostly attributable to a lack of knowledge of the nervous system's essential components: neurons and their functioning circuits. The treatment of a disease is dependent on determining the underlying cause of the disorder [3, 4].

In 1959, Nobel Prize-winning physicist Richard Feynman outlined the concept of nanotechnology, which he characterized as "making machine tools with the use of ever-smaller machine tools." Feynman further anticipated potential medical uses of nanotechnology, in which tiny robots, referred to as "nanosurgeons," are used to go through blood vessels and locate cardiac problems, whereupon they may then employ their nano-sized lancets to cut out the issue. Now, nanomedicine has many applications in areas, including basic research and medical practice, with the goal of helping people have better lives [5, 6]. Improved flexibility, precision, control, dependability, cost-effectiveness, and quickness are all provided by this tool. Nanotechnology methods are particularly well-suited for use in instances when fast treatments are required, such as in the treatment of cancer, the prevention of infection, and the regeneration of tissue [7, 8].

A few pathogenic processes of many central nervous system (CNS) illnesses remain unclear, and it is difficult to identify and treat these disorders [9]. One of the benefits of advances in nanotechnology is its ability to increase the specificity of complicated biological systems while also decreasing unwanted side effects[10–12]. These changes will have a large influence on neuroscience, particularly by allowing for the development of more effective and targeted therapies. The application of nanotechnology has the ability to assist in the transport of pharmaceuticals and small molecules across the blood–brain barrier, assist in maintaining neuronal function, and strengthen neuroprotective approaches, particularly those utilizing fullerene molecules [13–15].

**Fig. 1:**
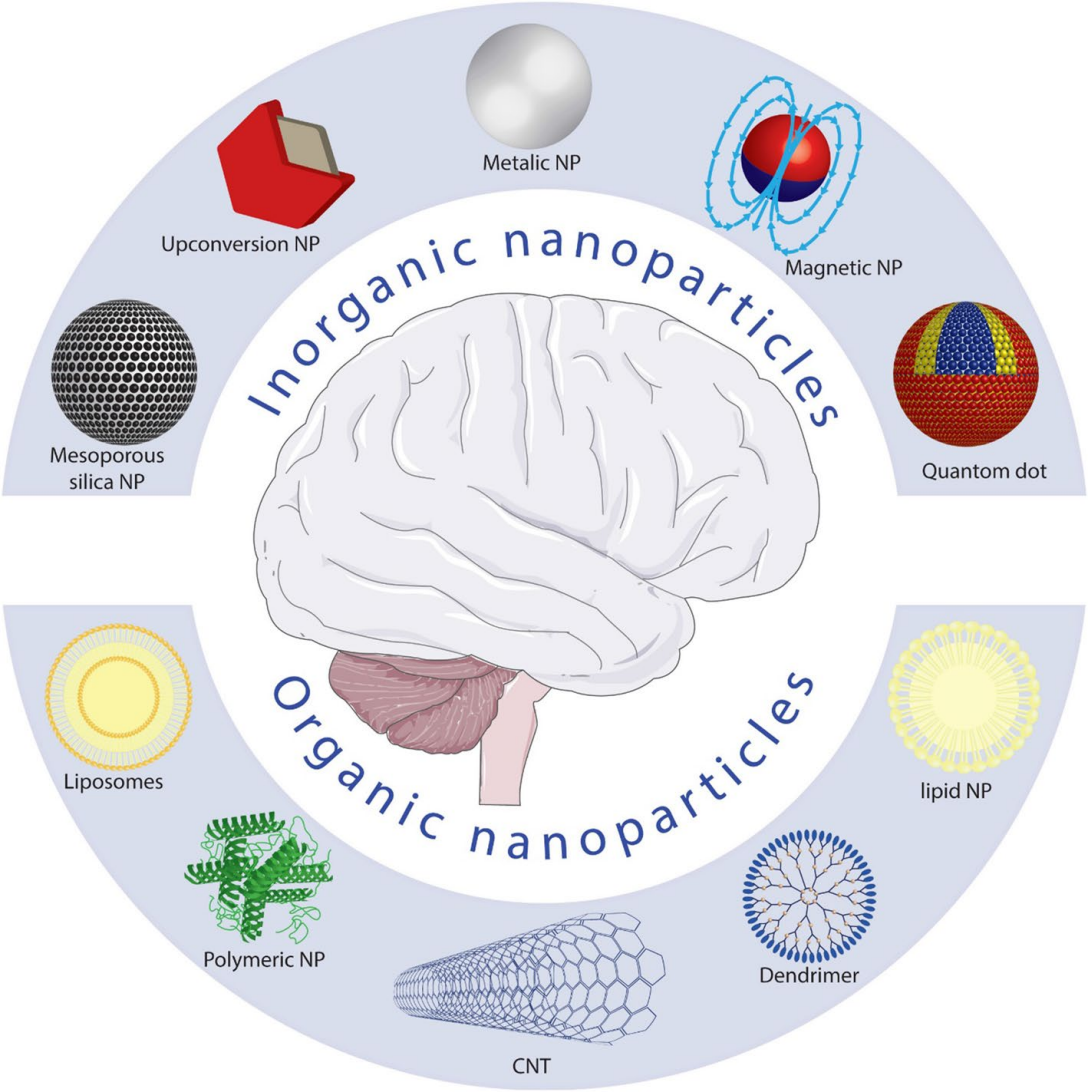
Neuroengineering nanoparticle toolkit. The anatomical configuration of the brain at various dimensions (top) and the various forms of organic and inorganic nanostructures that have been used in neuroscience are shown in this diagram

NeuroNanoTechnology is a novel therapeutic method in neuroscience that involves manipulating materials on a near-atomic scale to develop novel nanostructures featuring molecular, cellular, or atomic functionalities to control as well as repair damaged neural circuits [16]. Nanoscience includes the scientific discipline of materials at the nanometer scale. Thus, combining this field with neuroscience may help convert fundamental research into new materials and technologies for therapeutic intervention and surveillance for neurological disease conditions (Fig. 1) [17]. Nanostructures have exceptional chemical and physical characteristics, including durability, conductivity, strength, and chemical reactivity due to their small diameters, thus being extensively employed for electronics, sunscreens, cosmetics, and pharmaceuticals [18]. Nanoparticles have also opened up remarkable possibilities for biological applications. Nanostructures can also be inert, which makes them durable and allows them to attach to specific ligands, making them more effective during targeted therapy [19, 20]. Because of the difficulties of interfacing with neural cells or the mammalian nervous system, nanotechnology applications in scientific or clinical neuroscience are still in the initial phases of research. Considering this, a growing amount of evidence suggests that such innovations can contribute to neuroscience investigation [21].

This review highlights the wide range of nanotools and nanostructures that are presently being used, as well as the research that underpins their latest uses in neuroscience. This article presents an overview of existing technologies, improved imaging methods, and compounds intended to better associate with neural cells, as well as an explanation of the enormous effect that nanotechnology may provide on neuroscience investigations.

## Nanoneuroscience

Nanoscience is material engineering or nanosystem engineering, where the effective size is defined as 100 nm or less. It may be used to work with cell organelles and cellular components in diverse and little-known ways. It will be possible to make unique materials as we learn more about how matter and energy interact at this level [14, 22–24]. In contrast to conventional materials, nanoparticles with a diameter of 1–100 nm possess exceptional electrical, chemical, optical, mechanical, and magnetic properties; as a result, valuable nanosystems can be fabricated using these nanoparticles [25]. When compared to more established scientific disciplines, the field of nanotechnology is considered to be a more recent development. However, the present state-of-the-art relies on empirical approaches in materials science and engineering: smaller sized structures and devices created with these methodologies are able to show remarkable biological or cellular properties, while previously unknown mechanisms and interactions have been discovered [26, 27]. Richard P. Feyman is credited with laying out the basic concepts of the nanotechnology field in his seminal lecture titled "[There's] Plenty of Room at the Bottom," which took place in 1959. This year marks the beginning of the discipline of nanotechnology, which can be traced back to the year 1959. Nori Taniguchi was the first researcher to provide a definition of the term "nanotechnology" in the year 1974 [28]. The resulting remarkable breakthroughs have had an influence on the area of medicine, which has led to a requirement for attention from multi-disciplinary collaborations including material scientists, physicists, clinicians, and engineers.

The term "nanoneuroscience" refers to a branch of research that simultaneously addresses the primary objectives of the two distinct subfields of nanotechnology and neuroscience [29]. When nanotechnology is combined with neuroscience and bioengineering, it has the potential to transform fundamental research into innovative technologies and instruments for the diagnosis, therapeutic interventions, and surveillance of the pathophysiological conditions that are associated with neurological diseases [30]. The primary purposes of these advanced technologies are also to gain an understanding of the way the nervous system functions, in addition to how neurons interact with one another and start organizing themselves into arranged network systems in a variety of mental states or actions, to develop new treatments for illnesses that are associated with the nervous system [31].

Although it is still in its infancy, the collaboration between neuroscience and nanotechnology is already giving rise to innovative medical methodologies in the field of neuroscience [21]. Some of the broad principles that are being utilized include cell regeneration and cell protection; drug delivery; cell imaging; cell differentiation; and surgery. The incorporation of nanotechnology into piezoelectric effects as well as optogenetics is an additional indication of its future uses in the field of neurology [32–34]. These are only some of the potential applications of this union, which are not restricted to those that have been described above. In the end, the nanoneuroscience clinical translation shows that disorders of the CNS, such as neurodegenerative, psychiatric, and neurodevelopmental disorders, have the opportunity to be healed. On the other hand, the nanoneuroscience industrial translation suggests that there is a requirement for advancements to be made in brain–computer interface technologies [35].

Neuroscientists now have access to a greater number of opportunities than ever before because of the expanding fields of nanoneuroscience, which have lately opened the gate to a richer knowledge of neuronal functionality as well as the examination of its relationship to brain illness [36]. In contrast to the traditional methods used in the pharmaceutical industry for the creation and manufacturing of novel drugs, the emerging field of nanoscience has generated enormous optimism within the medical sciences [37]. The development of nanotechnology-based instruments that may be utilized in the prevention, diagnosis, monitoring, and treatment of pathological illnesses has also been made possible as a result of advances in nanoscience [38–40].

### The properties of nanomaterials for their application in neuroscience

CNS diseases are notoriously difficult to diagnose and treat due to the extremely protected nature of the CNS [41]. Because of the blood–brain barrier (BBB), bigger macromolecules are prevented from entering the brain [42]. Because of the limited anatomic accessibility, diagnostics and therapeutics are both more challenging in this diseased region than they are in other diseased sites. As a consequence of this, the therapies for CNS disorders using drugs that are administered systemically are frequently ineffective [43]. This issue is made even more difficult by the intricate functional and anatomical "wiring," the diverse molecular and cellular milieu, and the sophisticated processing of information [44]. As a reaction to these challenges, an expanding variety of groups are doing research into the characteristics of a wide range of nanoparticles to make use of the benefits that are inherently associated with their nanometer dimensions [45–47].

Nanomaterials, in comparison with their traditional, micrometer-scale equivalents, are capable of more precisely reflecting the surface features of organic tissues, such as energy and topography [25]. In addition, because of their diminutive size and the advances that have been made in the methods of synthesis, nanostructures have a wide variety of favourable characteristics. These characteristics include controlled release profiles, site-specific targeting or delivery, a high ratio of surface area to volume, adaptability in facilitating surface modification, and multi-functionality [48–50]. These characteristics might assist in improving the diagnostic process by increasing its sensitivity and resolution, reducing unwanted adverse reactions via focused therapy, particularly through the control of therapeutic value through the controlling of release of drugs in a specialized microenvironment. Consequently, nanostructures have the potential to be utilized as techniques for neuroprotective effects, as platforms for neuroimaging, as vehicles for the delivery of drugs, as scaffolds for neuroredifferentiation and neuroregeneration, and as instruments for neurosurgery [51]. In the nanoneuroscience field, a variety of nanomaterials, including inorganic and organic nanosystems, have been utilized as of late, and the possible uses of these nanostructures have been governed and analyzed (Table 1). Table 1 also provides a summary of the functional and structural features of these nanoparticles, as well as the prospective clinical uses of such nanostructured materials in the field of neuroscience.

**Table 1:** Functional and structural characteristics of nanomaterials, as well as the prospective uses of these properties in clinical neuroscience

Nanoplatform	Functional and structural features	Possible applicability in neuroscience	Refs.
Polymeric micelles	- Vesicles having an aqueous core are formed of a bilayer comprising lipids or phospholipids - Unilamellar or multilamellarAdjustable in terms of the magnitude of the synthesis: 20 to more than 500 nm - Modification and formulation of the surface are simple - Internalization of cells quickly while maintaining precise control over their release - Biocompatibility and a minimal likelihood of inducing an immune response	- Neuroprotectionl - Delivery of medications (including peptide drugs, such as thyrotropin-releasing hormone and DADLE (Tyr–D-Ala–Gly–Phe–D-Leu); Amphotericin B) to the central nervous system	[52] [53–55]
Lipid nanoparticles	- Surfactants provide stability for the solid lipid core lattice - Diameter: 10–1000 nm - Simple in regard to conjugation and functionalization Cytocompatibility	-Neuroprotection (Activation of P38 MAPK pathways and Bcl-2 family, diminution of the tunicamycin-induced endoplasmic reticulum stress upon internalization) - Gene silencing (siRNA (targeting the GluN1 subunit of the N-methyl-D-Aspartate receptor following intracerebroventricular and intracortical delivery; elucidation of the ion exchanger SLC26A11 as a voltage-gated ion channel engaged in neuronal swelling), mRNA for modulate mRNA splicing; oligonucleotide-loaded lipid nanoparticles)	[56–58] [59–61]
Nanoemulsion	- Water in oil: a water core that is kept together by surfactants as well as co-surfactants and is suspended in an oil media - Oil in water: oil droplets spread throughout an aqueous solution - Diameter: 20–200 nm	- Neuroprotection (down-regulation of amyloid precursor protein, total tau and phosphorylated tau, and β-secretase; preventing motor impairment and inhibition of complex I) - Drug delivery to CNS (Riluzole; glutathione and bromocriptine loading; tetrabenazine nanoemulsion)	[62, 63] [64–66]
Nanogel	- A hydrogel is made up of non-ionic and ionic polymeric materials that have been cross-linked - Diameter: < 150 nm - Modifications selectively applied to the surface - A high porosity level combined with a considerable loading capacity - Release profiles are both controllable and sustained	- Neuroprotection (such as a developed carboxyl-functionalized poly(N-vinyl pyrrolidone) nanogel system conjugated with for efficiently transported across the BBB in Alzheimer's disease; Methotrexate-loaded chitosan nanogels) - Drug delivery (Colloidal microgels; Magnetic nanogels to fluorescently labeled exosomes isolated from PC12 cells, enhancement of differentiation of adipose-derived stem cells to neuron-like cells)	[67] [68]
Nanocapsules	- A solid hydrophobic core enveloped by a monolayer of phospholipids - Diameter: 10–200 nm	- Neuroprotection (Triphenyl phosphonium coated nano-quercetin to moderate cerebral ischemia, preserving mitochondrial functional and structural integrity by sequestering ROS, modulating mitochondrial apoptotic cell death mediated by ROS) - Delivery of medications to the CNS (combining a icosahedral DNA-nanocapsule loaded with photoresponsive polymer with cellular targeting properties to cytosolic delivery of small molecules, such as dehydroepiandrosterone releasing)	[69, 70] [71, 72]
Gold nanoparticles	- Comprised of individual atoms of goldLow hydrodynamic dimensions: approximately 2.5 nm - Has a high surface area that is easily accessible, surface plasmon resonance, and RAMAN scattering - Modification as well as functionalization of the surface can be done easily - Durable and compatible with living organisms	- Drug delivery(Glycol-coated gold nanoparticles enhanced motor neuron survival, increased myelination of spared or regrown/sprouted axons) Labelling and nanoimaging (Due to the fact that the Se emission band is not located in close proximity to any other emission band and that the signal specificity is maintained in both methods of labeling, it was discovered that functionalized CdSe/ZnS quantum dots probes were ideal for use in nanoXRF(X-ray fluorescence); peripheral nerve nanoimaging)	[73–75] [76–78]
Iron oxide	- The minerals known as maghemite (Fe2O3) and magnetite (Fe3O4) - Superparamagnetic iron oxide (SPIO) diameter: between 50 and 150 nm - Ultrasmall SPIO diameter: between 10 and 14 nm - Has a high surface area - Because of its size, it can maintain circulation for longer and penetrate deeper into tissue	Erythrosine adsorption, labelling and nanoimaging ( magnetic resonance imaging was helpful for the localization of iron-oxide loaded macrophages in rat brains as a result of photodynamic treatment (PDT)-induced disruption of the BBB)	[79–82]
Quantum dots	- Crystals of colloidal semiconductors with a core of metalloid crystalline material - Can be covered with a variety of molecules or coupled with them - Dimensions: between two and ten nanometers - Superior photo- and chemical-stabilization - A high excitation coefficient at the molecular level - The possibility of breaking through the blood–brain barrier - Longer than average blood half-life - Lowest possible incidence of harmful reactions - Has a capacity to be ingested by phagocytic cells and removed from the body	- Nanoimaging (Quantum dots-labeled Aβ nanoprobes allow for the real-time observation of A aggregation, such as oligomerization and fibrilization, both in vitro and in intact cell systems; NIR light is utilized to stimulate cells inside the spectral tissue transparency window using a flexible quantum dot-based photovoltaic biointerface, colloidal quantum dots can be employed in wireless bioelectronic medicine for the brain) - Labelling (In primary neuronal cultures and in ex vivo rat brain slices, Quantum dot conjugated nanobodies are able to assess the kinetics of neurotransmitter receptors at excitatory and inhibitory synapses, respectively; Outgrowth and branching pattern of neuronal developments could be controlled by the use of the chemically modified element (nitrogen, boron, and phosphorous) doped carbon dots)	[83–86] [87–89]
Silica nanoparticles	- Silica nanoparticles are either nonporous or mesoporous, with a pore size of 2–50 nm - The presence of pores enables increased medication loading - Advantageous biocompatibility - Have an extremely high transparency - Materials that are dielectric (do not conduct electrons and do not absorb light)	- Stimulation of the growth of nerve cells and the development of neurites - Brain drug delivery(The survival rate of spiral ganglion neurons can be improved in vitro with the use of long-term release of brain-derived neurotrophic factor (BDNF) using nanoporous silica nanoparticles) - In vivo bio imaging and tracking (Dye-doped silica nanoparticles; functionalized manganese-doped silica nanoparticles effectively transports insoluble drugs to cross the blood spinal cord barrier)	[90] [91, 92] [93]
Carbon nanotubes	- Nanostructures the shape of cylinders constructed of graphene sheets wrapped upon themselves - High surface area - Diameter: from 1 to 4 nanometers - High surface area that is electrochemically sensitive (700–1000 m^2^ g) - Superior tensile and shear strength (elastic modulus ca. 0.64 TPa for an individual nanotube) - Superior thermal conductivity (particular multi-walled nanotube is greater than 3000 W rrr^−1^ K^−1^), excellent electronic flow (up to 10^9^ A cm^−2^), and low thermal expansion coefficient - Superior capacity for penetrating biological barriers	- Covering designed to enhance the electrical interaction for neural recordings as well as stimulation - For use in the process of neuroregeneration as scaffolds - Protein and DNA biosensors - Ion channel blockers Regenerative 3D scaffolds for the CNS (e.g., spinal cord and brain)	[94, 95] [96] [97] [98] [99]

The study of multifunctional nanoparticles is a relatively new scientific field that has undergone enormous expansion. These nanomaterials can be engineered to have a variety of particular capabilities and therapeutic applications [100, 101]. The vast variety of structures, wherein multifunctional nanoparticles can engage is recognized by the ever-expanding group of nanoparticles with one-of-a-kind thermal, mechanical, conductive, and toxicological characteristics [102]. Multifunctional nanoparticles might be non-porous or highly porous, filamentous or spherical, or any combination of these three forms. Although they can be made out of a broad range of substances and have many different architectures, multifunctional nanoparticles all adhere to the same core design principles [102]. An imaging area, such as a fluorescent probe, the molecules designed to target, such as targeted ligands capable of binding to expressed receptors on cells, as well as the molecules to be transported or released, such as a gene or medication, are all components that may be included in a standard multifunctional nanoparticle design [103]. These substances functionalize the nanoparticles, which is why they are called "multifunctional nanoparticles." These components can either be incorporated inside a porous lattice or have chemically attached ligands that rapidly start functionalizing during integration with the targeted systems [104]. Due to the vast number of internal structural configurations that are presently accessible and the vast number of physicochemical characteristics, as well as the multitude of structural possible variations that a provided nanoparticle could assume, multifunctional nanoparticles have the potential to potentially treat a wide variety of disorders in any physiological microenvironment in a manner that is cell-targeted and site-specific.

There is a factor-of-ten distinction to be made in half-lives between filamentous and spherical nanostructures, making shape a significant aspect that has a large influence on drug delivery for pharmacokinetic behaviour and penetrating the BBB [105, 106]. Therefore, the form of the nanomaterial is an essential consideration to make while selecting a vector. When nanoparticles are delivered into a microenvironment, they play an important role in determining the dynamic behaviour of interactions between nanoparticles and cells and, as a consequence, the cytotoxic risks of the nanoparticles [107]. Because it is probable that multifunctional nanoparticles will be administered into biosystems that already have pathological conditions, it is especially essential in the therapeutic setting to be knowledgeable of the interconnections between the environment and the nanoparticles [108]. Although the nanoformulation of the substance can have an effect on its structural qualities, the chemistry of the surface has a far greater effect on biochemical activity [109, 110]. Increasing our understanding of the pharmacokinetic and pharmacodynamic aspects of these interconnections may make it easier to create a nanoplatform for the next generation of technologies. They most likely rely largely on controlling neuronal development or otherwise modifying the differentiation of stem cells to influence the natural biological functioning directly.

### Carbon-based in neuroscience

**Fig. 2:**
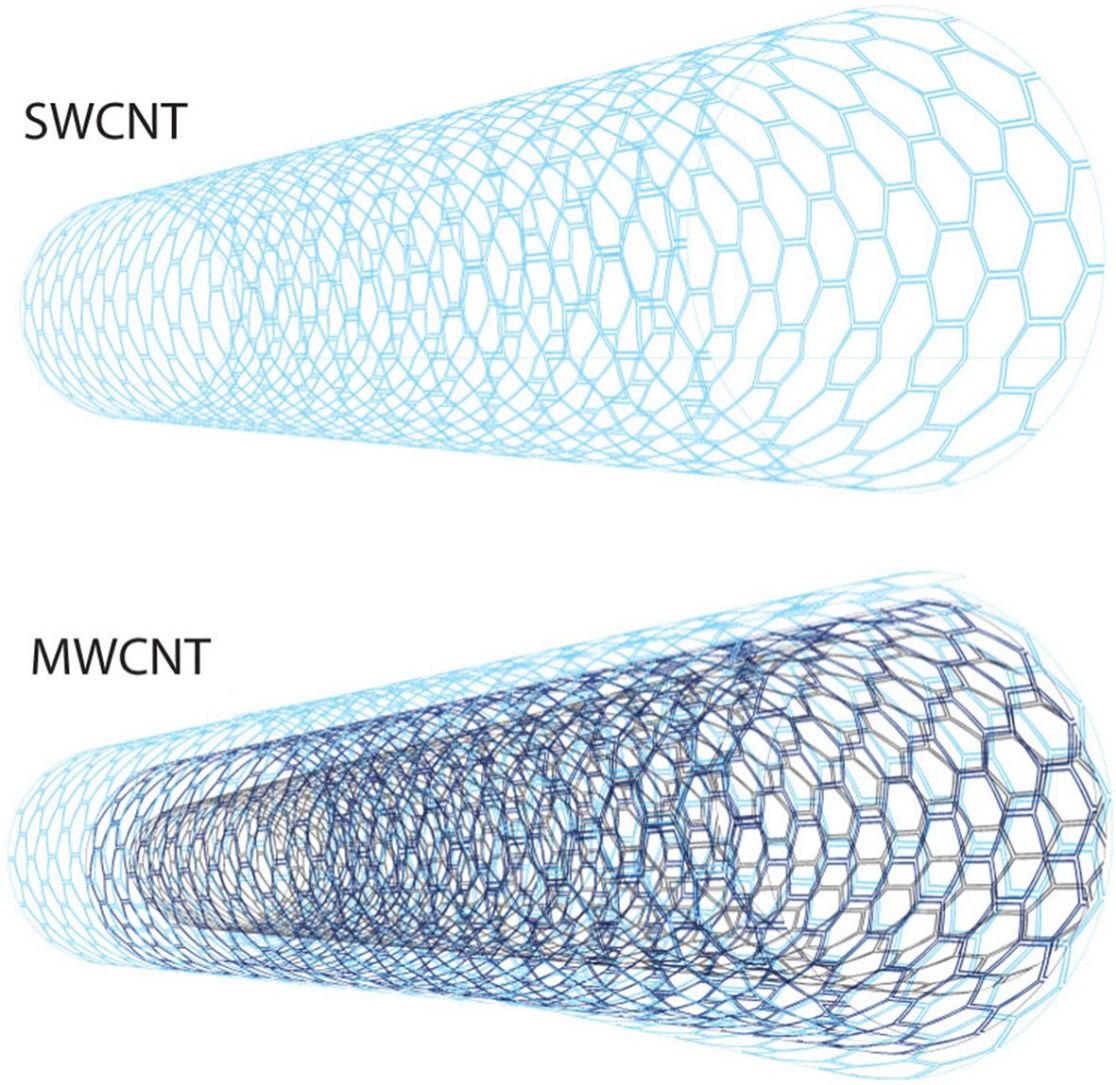
Graphical illustration of an SWCNT comprised of only one GF sheet, as opposed to an MWCNT comprised of multiple GF sheets

The continuous advancement of the substances employed to manufacture instruments, technologies, and scaffolds for application in nanotechnology-related disciplines is a crucial factor in the sustainable evolution of nanotechnologies. Carbon-based nanostructures, consisting of high-purity carbon with various atomic hybridization or geometrical patterns, must be given special emphasis in this context [111]. Until now, the three naturally existing allotropes of carbon (amorphous carbon, graphite, and diamond) have been accompanied by allotropes derived from synthetic methods (including carbon nanotubes (CNTs), graphene (GR), nanodiamonds, and fullerenes). GR and CNTs are presently the most prominent carbon nanostructures, which have been widely researched for their exceptional thermal and electrical conductivity, mechanical strength, and optical characteristics (Fig. 2) [112, 113]. Fluorination, for instance, serves to structure-insulate SWCNTs. Attaching RNA, DNA, antibodies, aptamers, and other biological probes onto biosensors allows for the specific capture of biological targets of importance [114].

A more frequent appearance of carbon-based compounds, notably CNTs and GR, has been seen in projects focused on both clinical and practical neuroscience [115–117]. In addition, it has been discovered that CNTs and GR connect or cross-link extremely efficiently with neural cells as a result of their unique physical and chemical characteristics. Until now, information regarding neural interactions with CNTs has often been more comprehensive than that of GF, which was revealed more than a decade later.

As a specific example, we can refer to CNTs, which have proven to be very effective in promoting neuronal growth [118, 119]. This early investigation discovered that the investigated substance improved both the proliferation and survival of hippocampus neuronal cells, as well as neurotransmission, with substantial increases in both self-generated action potentials and postsynaptic current flows. Furthermore, since that time, applications of CNTs for the purpose of cellular proliferation have grown and are being studied in greater depth. Studies have shown that CNTs are present at the single-cell stage, and this is believed to account for their effect on neurons, as they are known to have the ability to develop an electrical connection between neurones and their substrates, enhancing both glutamate and γ-aminobutyric acid (GABA) synaptogenesis as well as heterogeneous short-term synaptic plasticity [120, 121].

Future neuroprosthetics that use carbon compounds might use neuronal cultures grown on GF-based nanostructures that enhance neuronal development and signal transduction [122]. While there is initial interest in using it as a scaffold for tissue engineering, researchers are continuing to look at how it could benefit nerve tissue and whether it might promote tissue regeneration and brain function after damage [123].

In conjunction with the Mayo Clinic, carbon nanofiber-based (CNFs) nanoelectrodes have been developed as a neurochemical monitoring and stimulation instrument. Freshly formed ultra-small CNFs (CNFs with a size of 50 nm) were produced utilizing PECVD [124]. Since the electrical current or field is parallel to the wafer, the as-grown frameworks are free-standing and vertical. The CNFs and the exterior of the configuration have fractured walls, as shown by TEM observation (information collected but not seen here), and the subsequent deficiencies are suitable for the transfer of electrons.

To have electrical interaction between the measuring circuit and the CNFs, a wafer of silicon with a thermal oxide thickness of 500 nm is employed as the substrate, and the Cr or Pt thin layer is applied upon this substrate [124]. After that, a coating of nickel catalyst in the range of 10–30 nm is sputtered. At a temperature in the range of 600–700 °C and pressure in the range of 1–3 Torr, acetylene is being utilized to grow CNFs. On the wafer, the catalyst layer splits up into miniature droplets of varying dimensions, and growth on these catalyst particulates produces nanofibers. These non-coated CNFs can be considered triggering electrodes of any dimension, including a single electrode of CNF as small as 50 nm. Instead of a blanket catalyst layer that is sputtered, the catalyst can be patterned to produce CNFs of specific diameters at specific positions. To have structural performance, the space seen between nanofibers is packed and covered using SiO_2_. The top exterior surface of the CNF wafer is then polished chemically, leading to a smoother oxide surface including a few nanometers of CNFs Jutting outward. This implanted electrode can be used to measure neurochemical concentration levels [124].

Thin films of different synthetic substances, including Ge, Si, InP, GaAs, nitrides, oxides, and others, have facilitated advancements in optoelectronics, microelectronics, and microelectromechanical devices, among other fields. The majority of such substances have recently been developed as one-dimensional nanowires. As the semiconducting nanowire's radius becomes narrower than its Bohr radius, its bandgap energy tends to increase in comparison with the thin film's corresponding value. Nanowires have sparked interest in the development of sensor, electronic, photonic, and other technologies due to their remarkable thermal, optical, electrical, and other characteristics as compared to their bulk equivalents [125, 126].

### Magnetic-based tunneling junctions and magnetic nanostructures

Electrical and magnetic field gradients may have a spatial accuracy as small as sub-micrometers in the force-inducing nanostructures that control cellular activities, such as the deformation of membranes, the movement of organelles, or the migration of cells. Using magnetic nanoparticles, Gahl and Kunze induced neuronal cell function [127]. Magnetic tunnel junction (MTJ) technology was used to create the first bio-magnetic chip, which was proven to be biocompatible [128]. For MR cell imaging, it would be ideal to have probes that are both multifunctional and very sensitive to MRI, as well as highly efficient at labeling cells [129]. For brain progenitor cell (C17.2) MR imaging, Lu Zhang et al. developed fluorescent mesoporous silica-coated superparamagnetic iron oxide nanoparticles [91]. The size of the magnetic core was around 10 nm, while the size of the coating layer of fluorescent mesoporous silica was approximately 20 nm. A small proportion of these tagged cells were capable of being tracked as they migrated to the lesion areas using a clinical MRI scanner after these nanomaterials were implanted into the right hemisphere of stroke mice, which is diametrically opposed to the ischemic zone (3 T). Even more striking is the fact that the labelled cells could be watched as they homed in on the ischemia region even when they were delivered intravenously. Histological examinations of the brain tissues confirmed the findings of the MRI scans. They were quite useful for cell imaging and showed a lot of potential for MRI cell tracking thanks to their effectiveness. Theragnostic nanomedicine was created by Bingling Lin and colleagues to transfer superparamagnetic iron oxide nanomaterials and small interfering RNA/antisense oligonucleotides (siRNA/ASO) into neural stem cells [130]. This was done to inhibit PNKY long non-coding RNA (lncRNA). This nanomedicine not only prevents the neuronal development of neural stem cells by silencing the Pnky lncRNA, but it also makes it possible to detect neural stem cells in vivo using magnetic resonance imaging. The neuronal differentiation of neural stem cells is directed in this fashion. The better morphological and functional healing of the injured brain following a stroke was considerably helped by the accelerated neuronal differentiation of neural stem cells. The findings indicate that the multifunctional nanomaterials that target lncRNA have a significant amount of promise to improve stem cell-based therapeutics for the treatment of strokes. It has been demonstrated that when neural stem cells internalize magnetic nanobubbles (MNBs), which are assembled from magnetic nanomaterials, intramembrane volumetric oscillation of the MNBs causes an enhancement in intracellular hydrostatic pressure and cytoskeleton force, which ultimately leads to the activation of the Piezo1-Ca^2+^ mechanosensory channel [131]. This, in turn, activates the BMP2/Smad biochemical signalling pathway, which ultimately results in the differentiation of neural stem cells into neurons. The administration of low-intensity pulsed ultrasound can further expedite signalling that occurs via the Piezo1-Ca^2+^-BMP2/Smad pathway. This can be accomplished by applying an external shear stress force. In addition to this, magnetic resonance imaging and ultrasound imaging monitoring of neural stem cells that is based on MNB labelling can be used to give therapeutic results for neural stem cell therapy. The data obtained in vitro as well as in vivo reveals that a bubble nanostructure-induced physical force has the ability to tune and control the mechanical signalling system that is responsible for controlling stem cell growth. In the near future, new advances in the field of nanomagnetic fields applied to cell signaling, communication, and organization, as well as intracellular delivery, will be put to service in neurotherapeutic equipment.

### Liposomes

#### Liposomes containing GABA

Loeb et al. were the first researchers to describe using a GABA system that was encapsulated in liposomes [132, 133]. These researchers noticed a decrease in epileptic activity following intravenous injection (which lasted for 104 min), and they postulated that the liposomal transporter would increase the GABA penetration over the BBB. The formulation that was employed was designed using just natural phosphatidylserine as an ingredient. It is hypothesised that natural phosphatidylserine would result in the formation of vesicles that are less durable and more permeable, which will encourage the uptake of liposomes through macrophages [134]. In addition, essential aspects such as the magnitude and kinetics of release of the GABA were not investigated. In light of this, researchers recently examined the efficacy of an alternative liposome formulation for GABA release. Distearoyl-phosphatidyl–thanolamine–polyethylene glycol 2000 (DSPE-PEG2000), cholesterol (CHOL), and l-α-distearoyl-phosphatidylcholine (DSPC) were used in their production [135]. To encapsulate GABA at a level of 0.3 M in 0.9% NaCl, frozen and thawed multilamellar vesicles (FATMLVs) having a lipid content of 99 g/L were used. After completing all of the formulation procedures, we found that the encapsulation rate of GABA had attained an average effectiveness of approximately 30%. To produce liposomes with very reduced membrane permeability, high-phase transition temperature phosphatidylcholine was combined with cholesterol to form the membrane of the liposomes [136]. Furthermore, in vitro data suggest that GABA is released from liposomes at a relatively sluggish rate, with just 60% of the GABA being released following 5 days of incubation at 37 °C. Other significant features of these liposomes include their average size of 200 nm, the inclusion of a pegylated lipid, which helps to slow down the rate at which cells are capable of capturing them through endocytosis or phagocytosis, and the cell-mediated drug release that is encapsulated within them [137].

In a study, designers used an in vitro model to show that neurons that are subjected to GABA-containing liposomes for a period of 24 h undergo two significant alterations at the molecular level. First, there has been a considerable rise in the number of GABAA receptors (by 50%). A significant rise in levels of nitric oxide (NO), which is an essential component of the intracellular communication that occurs in the CNS. The decrease in protein inhibitor of neuronal NO synthase (PIN) might be the cause of the rise in NO [138]. The binding of PIN results in the instability of active neuronal NO synthase dimers, which in turn leads to the generation of functionally hindered and catalytically inactive monomers, which in turn results in a reduction in NO generation [138]. It is possible that the stabilisation of neuronal NO synthase dimers within neurons subjected to liposomes containing GABA is the cause of the significant rise in NO levels [138].

#### Addressing challenges such as the BBB and microglial reaction

The viewpoint of using liposomes containing GABA for targeting diseases of the CNS must take into consideration two preponderant difficulties, namely, passing the BBB and the reaction of microglia. This is obvious of any medication delivery nanosystems for reaching the brain. The BBB safeguards the brain from potentially dangerous blood-borne pathogens, but it also restricts the administration of a huge number of medications used to treat neurological illnesses [139]. There is no doubt that one of the most important things that has to be done right now for the advancement of nanoscience as it relates to the nervous system is the pursuit of diverse BBB delivery techniques.

After intravenous injection, long-circulating nanomaterials have the potential to passively cross the BBB in the context of the treatment of disorders that undermine the BBB's integrity [139, 140]. The integrity of the BBB is frequently compromised by brain tumours, and this deficit can also be the result of other brain illnesses [141]. To improve the transport of drugs via liposomes, another technique that has been attempted is to temporarily open the BBB using focused ultrasound or osmotic (mannitol) shock [142]. To improve the mechanism, magnetic nanomaterials were also paired with a magnetic field that was induced in the brain [143].

The liposomes that were shown to be the most successful at penetrating an intact BBB include cationic vesicles and liposomes with surfaces that were functionalized by targeting ligands [144, 145]. These ligands precisely bind to receptors or transporters that are expressed on the endothelial cells' membranes in the brain. The intraarterial injection of cationic vesicles was discovered to be more successful at depositing liposomes into the brain than either anionic or neutral vesicles. This may be because of the electrostatic interactions between the negatively charged cellular membranes and the cationic liposomes, which enhanced nanoparticle uptake by adsorptive-mediated endocytosis [146]. However, the application of cationic nanovehicles for the transport of pharmaceuticals into the brain is restricted by nonspecific absorption by peripheral tissues in addition to their attachment to serum proteins, which reduces the surface charge of the nanosystems. To achieve therapeutic effectiveness, then, enormous quantities of these nanovehicles will be necessary; yet, these nanostructures have the capacity to be cytotoxic [147].

Utilizing the many transporters and receptors that are situated at the BBB, transcytosis mediated by a receptor is one of the most efficient methods for passing through the BBB [148]. The low-density receptor-related lipoprotein is an example of this type of scenario. Other molecular targets, such as the insulin receptor, the transferrin receptor, and the glucose transporter GLUT1, have also been utilised effectively [149, 150].

Bypassing the BBB has been extensively investigated through the use of non-invasive delivery methods, such as mucosal or ocular delivery [151, 152]. For instance, intranasal injection is a feasible method for delivering medications to the brain, and studies have shown that cationic liposomes are particularly efficacious when delivered in this manner [153, 154].

The introduction of liposomal formulations into certain areas of the brain can also be accomplished through the use of invasive procedures [155]. Passing the BBB in humans with the use of invasive procedures that involve intraparenchymal or intracerebroventricular straightforward administrations is thought to be far from optimal due to the necessity of hospitalisation, the potential for scarring of brain tissue, and the possibility of infectious disease [156, 157]. Direct administration, on the other hand, provides the opportunity to bring the therapeutic into play locally, therefore, lowering the risk of systemic toxic effects and protecting the healthy tissue in the surrounding area [158, 159]. When considering how to treat some neurological diseases, direct administration should be considered a viable option, because it is a genuine possibility. In this regard, the direct administration of dopamine-containing liposomes into the striatum enhanced localized extracellular levels of dopamine over a period of 25 days, which resulted in a reduction in the deficiencies related to a mouse model of Parkinson's disease [160]. In a model of brain tumour seen in rodents, direct administration of liposomes into the brain by convection-enhanced transport was investigated. According to the findings, liposomes were capable of efficiently distributing themselves throughout the tissue of the tumour, which provides a foundation for therapeutic applications to targeted sites of interest [161, 162]. Finally, direct administration of liposomes into the CNS seems to be a viable method for therapeutic targeting of certain brain areas.

After entering the CNS, the next challenge is how the microglia will respond to the liposomes they encounter [163]. Microglia are the primary immunological cells of the neurological system. They serve as a line of defence against the invasion of the CNS by pathogens that enter the body through the bloodstream or through traumatic damage to the neural tissue [164, 165]. Microglial activities cluster toward the site of injury, creating a barrier between normal tissue and damaged tissue. The release of inflammatory cytokines occurs as cells migrate to the location of the damage. It appears that ATP, which is produced from injured tissue, is responsible for regulating the chemotactic responses. Microglial reactivity leads to the release of a wide variety of chemicals, including lipid mediators, free radicals, and cytokines, which are all implicated in the mechanisms of inflammation and tissue healing [166, 167]. It is fascinating to note that there is data that suggest that the inflammation activity that occurs as a result of microglial activation can be suppressed by a liposomal ingredient [164]. Research has shown that microglia have the ability to preferentially bind liposomes that are richer in phosphatidylserine [168, 169]. Phosphatidylserine, by its interaction with particular phosphatidylserine receptors, inhibits the typical activating of macrophages that leads to pro-inflammatory responses [170]. Because of this, it has been demonstrated that liposomes that contain phosphatidylserine can limit the production of pro-inflammatory cytokines from microglia and can prevent the activation of the mitogen-activated protein kinase p38, which in turn can suppress pro-inflammatory activity in microglial cells [171]. In addition, Hashioka and colleagues demonstrated that liposomes containing phosphatidylcholine and the phospholipid phosphatidylserine suppress the activation of microglia, which results in the liposomes possessing both antioxidative and neuroprotective capabilities. These findings suggest that the liposomal formation, in and of itself, is capable of modulating the inflammatory reactivity of microglia [172, 173]. This is a beneficial property to take into account when contemplating the use of liposomes for neurodegenerative diseases or other conditions affecting the CNS.

### Polymeric nanoparticles

Due to their controlled drug release, programmable architecture (10 to 1000 nm), biocompatibility, and non-toxicity, polymeric nanoparticles in particular represent a viable option as a drug delivery system for CNS targeting [48, 174]. These polymeric nanomaterials are easily modifiable with certain ligands that bind the endothelial cells' receptors; as a result, the effectiveness with which transcytosis occurs is increased [175]. In addition, polymeric nanomaterials exhibit a longer circulation duration than other nanoparticles and are capable of biodegradation [176]. Following the process of cellular absorption and internalisation, the polymeric lattice has the potential to be activated to release the medication, producing a therapeutic impact that is sustained, targeted, and protected [177, 178]. Polymeric nanoparticles are flexible enough to be capable of delivering a broad variety of medications, for instance, through interactions that are hydrophobic, hydrophilic, or electrostatic, as well as reactive covalent bonds [179].

#### Synthetic polymeric nanoparticles for BBB transfer

##### Poly(Alkyl Cyanoacrylate)

Poly(alkyl cyanoacrylate) (PACA), also known as superglues, are a kind of polymer that has been utilised extensively in the medical field as a suture material [180]. In 1972, Couvreur and colleagues were the first to produce PACA nanomaterials [181]. They have a limited potential for toxic effects and are decomposed by esterases that come from pancreatic juice and are thus found throughout the intestinal tract (when taken orally) or through serum esterases that are found throughout the bloodstream [182]. The amount of time it takes for the substance to degrade is measured in hours and may be altered by changing the length of the alkyl side chain. For instance, polymeric materials having a longer side chain (such as octyl) degrade at a slower rate than those with a shorter side chain (such as butyl) [183]. In addition to this, the selection of side chains has an impact on the overall toxicological profile [184]. PACAs can be produced through a variety of polymerization processes, including zwitterionic polymerization, anionic polymerization, and free radical polymerization [185]. PACA nanomaterials can be manufactured through polymerization in an acidic medium phase or through interfacial emulsion polymerization. The cyanoacetic acid in PACA nanomaterials can be esterified with other substances, such as pharmaceuticals, folic acid, or polyethylene glycol (PEG)-amine, to produce cyanoacetate esters, which can then be polymerized. This process is one way to functionalize PACA nanomaterials. Through encapsulation or adsorption, several medications, such as weakly soluble or hydrophilic compounds, proteins, nucleic acids, and peptides, have been loaded [186–189]. PACA nanomaterials have been modified using PEG to prevent their absorption by macrophages and with polysorbate 80 to strengthen their capacity to permeate the BBB to be used for brain administration [190, 191]. In a separate piece of research, PACA nanomaterials were coated with an anti‐Aβ1–42 antibody [192]. This led to a large increase in the amount of Aβ that was found inside the plasma, which in turn led to memory recovery in a mouse model of Alzheimer's disease. In fact, a number of PACA-formulated nanomaterials have been the subject of investigation in clinical studies, although not specifically for CNS illnesses [193–195]. For example, patients with resistant solid tumours or patients with hepatocellular carcinoma have been evaluated using PACA nanomaterials packed with doxorubicin or mitoxantrone, respectively [196, 197]. Due to significant acute respiratory distress episodes, a phase II study had to be terminated; nevertheless, this problem was resolved by switching the delivery modalities from an intrahepatic artery route to an intravenous route with slow administration [198]. In contrast to the highest standard of treatment, a phase III trial unfortunately failed to demonstrate any additional survival advantage for participants. It has been hypothesised that the variable drug encapsulation percentage and release patterns are one of the reasons why there has not been enough progress made in clinical translation [199].

##### Poly(Lactic‐*co*‐Glycolic Acid)

Poly(lactic-*co*-glycolic acid) or PLGA is a class of linear copolymers that are able to be manufactured by combining lactic acid and glycolic acid in a variety of proportions [200, 201]. These proportions determine the structure of the finished product. The Food and Drug Administration (FDA) of the United States has given its blessing for the use of PLGA in a variety of medical applications, including drug delivery systems, sutures, and screws as biomaterials [200, 202]. The PLGA copolymers are biodegradable and non-toxic by a process called hydrolytic de-esterification, which is then accompanied by the removal of their monomeric anions, which are lactate and glycolate [200]. Changing the ratio of lactic acid to glycolic acid allows for fine control over the level of crystallinity, mechanical strength, degradation rate, and consequently drug release and encapsulation kinetics. Because of its methyl sidechains, poly(lactic acid) (PLA) is a crystallized hydrophobic polymer, whereas poly(glycolic acid) (PGA) is hydrophilic and stiff and has a poor mechanical strength [203]. Therefore, PLGA copolymers featuring a larger ratio of PLA:PGA are more hydrophobic, and as a consequence, they have a slower rate of degradation in addition to a slower rate of medication release. For instance, a 50:50 mixture biodegrades in about a week (depending on its molecular weight), but pure PLA can degrade in up to 18 weeks [200].

The synthesis of PLGA is possible through the use of a few different methods, including the Segmer assembly polymerization, ring opening polymerization, and polycondensation process [204, 205]. Using methods of soft lithography, it is also possible to manufacture non-spherical nanoparticles, such as those with a cylindrical form [206]. Surface changes can be made by means of the terminal carboxylic acid groups, for instance, by producing triblock (PLGA–b-PEG–b-PLGA) or diblock (PEG–b-PLGA) copolymers, or by inserting targeted moieties, such as antibodies or folic acid [207, 208]. As a consequence of this, different types of medicinal molecules, such as anti-inflammatory medicines, proteins, antibiotics, and chemotherapeutics, have been encapsulated inside of PLGA nanoparticles [209]. Many formulations of PLGA have been investigated for their ability to pass the BBB [210–212]. In transgenic mice, administration of PLGA nanoparticles coated with a cyclic peptide targeting the transferrin and packed with a curcumin and inhibitor peptide resulted in significant enhancements in recognition and spatial memory [213]. In addition, two non-CNS preparations targeting PLGA have been given the green light for use in clinical trials. In 2006, Genexol-PM was granted approval for the treatment of breast cancer as well as head and neck cancer in South Korea, while in 2007, Nanoxel was granted approval for the treatment of many types of cancer in India. In addition, phase II clinical studies targeting a prostate-specific membrane antigen for prostate cancer employing PGLA nanomaterials packed with docetaxel (BIND‐014) were effectively completed in 2016 [183].

##### Polyamidoamine dendrimers

Dendrimers are biodegradable, three-dimensional polymer macromolecules that feature a centralized core that is surrounded by a corona that contains reactive functional groups [214]. Because of the layer-by-layer construction process that is used to create them, their dimensions are expressed in terms of generations [215]. There are several distinct varieties of dendrimers, the most notable of which is founded on polyamidoamine (PAMAM). The synthesis of PAMAM can be carried out in either a convergent or divergent manner by employing Michael addition processes, which are then followed by amidations. Other surface functional groups, such as carboxylic acid (COOH) or hydroxyl (OH), can also be introduced into the material in addition to amines [216, 217]. These functional groups have the potential to increase the water solubility of PAMAM dendrimers, restrict their capacity for opsonization, and decrease their rate of removal via the mononuclear phagocyte system (MPS)[218]. PAMAM can load pharmaceuticals either through the process of physically entrapping them in the hydrophobic holes or through the process of conjugating them to the functional groups on the surface [219]. Because PAMAM dendrimers are typically smaller than 15 nm (based on generation)[220], researchers have investigated the possibility of using them as an alternative potential drug delivery method for the brain. For example, it has been demonstrated that dendrimers are capable of crossing the blood–brain tumour barrier (BBTB) in mice suffering from neuroinflammatory diseases, such as cerebral palsy [221], malignant glioma [222], and traumatic brain injury [223]. Furthermore, it was demonstrated that PAMAM dendrimers of the third generation, which were encrusted with a streptavidin adapter, could transfer an undamaged BBB via transcytosis. Furthermore, gently protonated G4 PAMAM dendrimers, which contained 10% amine, were capable of passing through the brains of healthy mice [224, 225]. In spite of the high clinical hopes and research efforts, there has been a limited amount of clinical translation for dendrimers. The sole polylysine dendrimer-based antibacterial therapy that has been authorised for use in healthcare items is manufactured by Starpharma. Despite this, there have been efforts made to dramatically reduce dendrimer synthesis mechanisms and optimise the particulate configuration [226]. For instance, multiple functional groups have been modified, and the integration of internal structure functionalization has been achieved, so that a higher medication loading can be fulfilled.

#### Natural polymeric nanoparticles

##### Alginate

The brown seaweed that is used to make alginate is a straight, unbranched polymer that has an anionic charge (phaeophyceae). It is a randomized copolymer that is composed of α‐l‐guluronic acid and β‐d‐mannuronic acid linked together by 1,4-glycosidic connections [227]. Alginate is a non-immunogenic chemical that has been authorised by the FDA and has been employed for applications including tissue engineering, medication delivery, and wound healing [228]. Utilizing the carboxylic acid and hydroxyl functional groups that alginate possesses allows for the introduction of highly reactive functional groups (for example, aldehyde groups), as well as biochemical (for example, amino acid) groups or chemical (for example, phosphate or sulphate) groups that can significantly raise the biointegration and bioaffinity characteristics of the alginate [229]. The complexation of alginate, employing divalent cations or cationic chemicals, including Ca^2+^, is the method that is used to produce alginate nanoaggregates and nanocapsules [230]. An emulsion of water and oil is used in the production of alginate nanospheres, which is followed by a gelation step [231]. Mixing alginate to other polymeric materials, such as poly[(2-dimethylamino) ethyl methacrylate], or using disulfide cross-links are also viable options for synthesising responsive alginate nanomaterials in terms of redox or pH[232]. Recent research has revealed successful transport of nanoparticles made with alginate to the brain. For instance, it was demonstrated that alginate–cholesterol micelles covered with lactoferrin were capable of transporting a neuroprotective steroid into the brain, and it was demonstrated that alginate nanomaterials cross-linked to chitosan were capable of enhancing the brain shipment of an antidepressant [233, 234]. In addition, doxorubicin–alginate nanocomplexes containing chitosan frameworks showed increased absorption into the rabbit brain [235].

##### Chitosan

Due to its affordability, biodegradability, and accessibility in a variety of molecular weights, chitosan, a cationic linear polysaccharide, is one of the most extensively employed natural polymeric nanomaterials for drug delivery [236, 237]. In addition to this, it possesses a variety of exceptional intrinsic biological capabilities, such as antibacterial, antitumor, and antioxidant capabilities[238]. Chitosan is produced by the partial *N*-deacetylation of chitin, a naturally occurring polymer that can be collected from fungi or crustaceans [239]. Chitin contains randomly dispersed *N*-acetyl-d-glucosamine and β‐(1,4)‐linked d‐glucosamine units. Chitosan has three different kinds of functional groups, which can be used for a variety of different chemical modifications [240]. These functional groups include primary hydroxyl, amine, and secondary hydroxyl [241]. The molecular weight, the degree of deacetylation, and the chemical changes can be changed to alter the biodegradability of the substance [238]. Chitosan nanomaterials can be produced using a number of different processes, some of which include chemical cross-linking, ionic gelation, and microfluidic synthesis [242, 243]. Because of their positive charge, which boosted cell absorption and made them amenable for pairing with negatively charged therapies, these natural nanomaterials have shown promise in brain delivery. For instance, PEG–chitosan nanomaterials modified with antibodies demonstrated high brain absorption, which researchers believe is due to the complementarity between the antibody and the positively charged chitosan [244]. However, chitosan nanomaterials have certain drawbacks, including poor management of their molecular weight and the limited drug loading effectiveness of hydrophobic materials. In fact, it has been demonstrated that the effectiveness of drug loading may be increased by the application of chemical changes, such as grafting palmitic acid [230, 245].

### Micelles

Micelles, which are vesicles that are composed of amphiphilic copolymers (polymeric micelles) or amphiphilic surfactants (non-polymeric micelles), have lately captivated the attention of researchers as a potential drug carrier route to the CNS [246]. Polymeric micelles are thought to be more durable than non-polymeric micelles, because they have a lengthy action time and good biodistribution [247]. They feature a core–shell structural architecture with a diameter varying from 10 to 100 nm, comprising of an outside hydrophilic environment that is usually composed of PEG as well as an interior hydrophobic core that is manufactured by means of molecules, such as fatty acids, phospholipids, polypropylene glycols, and polycaprolactone; hence, they enable the loading of hydrophobic pharmaceuticals [248]. The exterior hydrophilic coating gives micelles durability throughout an aqueous environment, extends the time that they spend travelling through the bloodstream, shielding it from the reticulo-endothelial system (RES), and further facilitates their aggregation in specific areas with leaky vasculature [249]. The category of pluronic (also known as Poloxamers) block copolymers is of particular interest due to their ability to suppress drug efflux transporters (for example, P-gp efflux transporters, which are abundantly expressed on the BBB) and increase drug delivery to the CNS [250]. In addition to this, it has been established that they improve the stability and solubility of the medication in plasma, which in turn makes it easier for low-molecular-mass pharmaceuticals that are integrated into them to be transported to the brain.

There have been a plethora of efforts made to modify the micelles in such a manner that an increased concentration of packed medicine may readily pass over to the opposite side of the BBB. One such modification involves targeting the receptor at the luminal side of the BBB with polyclonal antibodies against α2-glycoprotein, a brain-specific antigen, or insulin. After loading these modified micelles with a fluorescent dye or the neuroleptic medication haloperidol, intravenous injection of these micelles into mice led to improved transport of the luminous dye toward the brain as well as a significant enhancement in the neuroleptic impact of haloperidol [251, 252].

The pharmaceutical molecule is directly conjugated, and the targeting moiety is attached to the amphiphilic region, which is another variation of the micelle method. For example, Zhang et al. conducted research on a transferrin-modified cyclo-(Arg–Gly–Asp–d-Phe–Lys)–Paclitaxel conjugate-loaded micelle. Their findings showed an enhanced uptake by brain microvascular endothelial cells in vitro, as well as an extended retention in glioma tumors in vivo, without observing any significant toxicity [253]. Chitosan oleate self-assembled polymeric micelles and PLGA nanomaterials coated with CS–OA, which gives a positive surface charge, were produced and examined for their interaction with Caco-2 and HeLa cells. PLGA–CS–OA was found to be more stable when compared with polymeric micelles; however, micelles did not show any significant difference in stability [254].

### Solid–lipid nanoparticles

These days, solid–lipid nanoparticles (SLNs), are garnering a significant amount of interest as potential innovative drug transporters. They are also at the vanguard of the fast-developing nano-delivery system [255]. These are aqueous colloidal nanocarrier systems that are made of physiological lipids (fatty acids, steroids, triglycerides, and waxes, etc.), which are distributed in water or in an aqueous surfactant solution, and have the capacity to become solidified upon cooling [256]. There have been a number of attempts made to improve the capacity for loading drugs and the long-term durability of SLNs. One of these attempts was the creation of nanostructured lipid carriers (NLCs) through the blending of solid lipids into liquid lipids or the combination of spatially dissimilar lipids [257]. Research that compared SLNs and PEG-modified SLNs packed with anticancer medications such as camptothecin and doxorubicin indicated that the modification of SLNs with PEG improved their ability to penetrate the BBB and improved the transport of pharmaceuticals to the CNS [258, 259]. Because of their low inherent physical durability, cytotoxic effects, protection of labile medicines from degradation, and regulated release, SLNs have a greater potential than polymeric nanoparticles to be employed as a brain therapeutic delivery platform, particularly for the treatment of brain tumors [260]. Endocytosis, a process that occurs inside the endothelial cells that line the blood capillaries in the brain, transcytosis, or penetration via the tight junctions that exist between endothelial cells are all potential mechanisms that might be responsible for their distribution across the BBB [261, 262]. In addition, the adsorption of a plasmatic protein onto the surface of SLNs, such as apolipoprotein E, might make it easier for the protein to be taken up into the brain [263, 264]. This would be facilitated by adhesion to the endothelial cells that make up the BBB. To obtain target-specific delivery of medications across the BBB, the technique described above has been utilised for the encapsulation of a wide variety of pharmaceuticals. After intravenous and intraduodenal injection, it has been shown that sterylamine-based SLNs carrying clozapine, an antipsychotic medicine, can effectively deliver the pharmaceutical into the brain [265]. Additional examples of drug-laden SLNs are atazanavir-packed SLNs for the treatment of HIV encephalitis and quercetin-loaded SLNs for the treatment of Alzheimer's disease [266, 267]. A study has shown that riluzole-loaded SLNs are more effective than free riluzole in a rat model of amyotrophic lateral sclerosis (ALS) that was established by vaccination with experimental allergic encephalomyelitis [268].

## Biological sensors

Biosensors include instruments that have an exterior surface on which the probe-target association can take place and then convert the interconnection into an observable signature [269]. The pulse quality and intensity can involve many different types of signals, including cantilever deflection, optical, electrochemical, and electrical signals. A biosensor, as described by the IUPAC (International Union of Pure and Applied Chemistry), is "an instrument that further detects chemicals using unique biochemical reactions regulated through immunosystems, isolated enzymes, organelles, tissues, or whole cells, typically by optical, thermal, or electrical signals" [270]. Optical technologies are now well-evolved and demonstrated to be able to recognize single molecules [271]. The fluorescent markers (for instance, dye) become attached to the biological recognizing substances or probes, resulting in a fluorescent signature from the probe-target association. The magnitude is proportional to both the concentration thresholds and the potency of which the desired compounds are captured. Because labeling is time-consuming, optical label-free strategies have been developed [272].

Electrical-based biosensors can be made using bio-field-effect transistors (BioFETs), which have a reference electrode instead of the traditional transistor and a liquid gate [273, 274]. In comparison with the operation of the non-modified gate, the current–voltage (I–V) properties of the gate would change if a probe was connected to it. The I–V curve will shift proportionally to the concentration levels of specified target molecules in the solution as the probe-target association progresses. BioFETs have been studied widely for the identification of multiple biological markers throughout the biomedical and environmental surveillance areas [275, 276].

Electrochemical strategies that use metallic materials (Pt, for instance) or carbon electrodes have become another type of electrical transduction. Carbon electrodes have long been used in the form of glassy carbon, graphite, diamond, or carbon paste; more recently, GF and CNTs have gained popularity [277]. These carbon-based electrodes are usually employed in potentiometric, amperometric, or impedimetric modalities, where detectors with affinity or selectivity for the targets of concern are functionalized onto the electrodes. RNA, DNA, aptamers, antibodies, and other probes are all possible.

### Detecting neurotransmitters

The primary regulating molecule in the brain is the neurotransmitter, which allows the brain's neurons to function as well as guide human physiology and behavior. They take the information that is stored in various parts of the brain and transmit it across the neurones to carry out the tasks needed [278, 279]. Nor-epinephrine (NE), epinephrine (EP), glutamate, acetylcholine (ACh), and serotonin or 5-hydroxytryptamine (5-HT) are all considered excitatory neurotransmitters. In addition, glycine and GABA are all considered inhibitory neurotransmitters, while dopamine (DA) may have both inhibitory and excitatory effects [280, 281]. Many aspects of the human body, such as temperament, sleep, appetite, learning, feelings, memory, attentiveness, and a number of other cognitive activities, are regulated by broad ranges of neurotransmitters. Alzheimer's disease, epilepsy, traumatic brain injury (TBI), and Parkinson's disease, have all been shown to be related to unusual neurotransmitter concentrations [282–284]. In addition, there is some evidence to suggest that abnormally high levels of neurotransmitters are connected with substance use disorders and have been linked to life-threatening pharmaceutical responses [285, 286].

Parkinson's syndrome is believed to be caused by a lack of DA, whereas schizophrenia is assumed to be influenced by an accumulation of DA [287]. 5-HT concentrations have also been attributed to conditions, such as depression and addiction. In an environment of ascorbic acid (AA) containing 100–1000 fold greater concentrations, both HT-5 and DA are found in minute amounts. The majority of the detection strategies utilized throughout the literary works and discussed here, particularly electrochemical methods, provide a quick reaction time benefit, but the true difficulty is AA subjectivity between HT-5 and DA. The oxidation capabilities of the two neurochemicals are just 150 millivolts apart. As a result, several traditional electrodes struggle to produce an extremely high diffraction peak between the two objectives, particularly if both are available in AA near appropriate concentration rates. As a result, electrodes made of nanomaterials have been developed, as have advanced electrodes with nanostructured coatings [288–295]. Nanomaterials have a large electroactive surface area and, therefore, can enhance electron transport between the molecules being targeted and the electrode surface. This eventually results in an increase in the electrical sensing platform's sensitivity. It has been claimed that other benefits, such as strong electrical mobility, electrical conductivity, and excellent electrocatalytic characteristics, can improve both sensitivity and selectivity with regard to DA detection [296]. To be more precise, carbon-based nanomaterials, such as CNTs and GF derivatives, have the capability of absorbing DA via a–b stacking and augmenting DA-specific signals thanks to the superior electrical characteristics they possess[297, 298]. Beside that, CNT and polymeric composites have been produced for use as implantable neural electrodes. An electrical impedance that is 15 to 20 times lower than that of a conventional platinum–iridium (Pt–Ir) wire was achieved by insulating individual carbon nanotube fibers using a polystyrene–polybutadiene copolymer [299]. In addition, the microelectrodes' ability to bend and their small diameter make them more biocompatible. This is because they better integrate with neural tissue, which leads to less delamination when they are implanted throughout the brain [300]. Implantation into the subcortical regions of the brain of a rat model served as the testing ground for this hypothesis. In the rat model of Parkinson's disease, administering deeper brain stimulation employing CNT fiber electrodes led to a reduction in the severity of the illness's motor symptoms. Six weeks following the implantation, the brain tissue as well as the electrode were examined, and they showed a decreased inflammatory reaction in comparison with Pt–Ir electrodes. In addition, neuronal activity was measured in the primary motor cortex of rats over the course of 4 weeks, revealing just a little decline in signal strength.

**Fig. 3:**
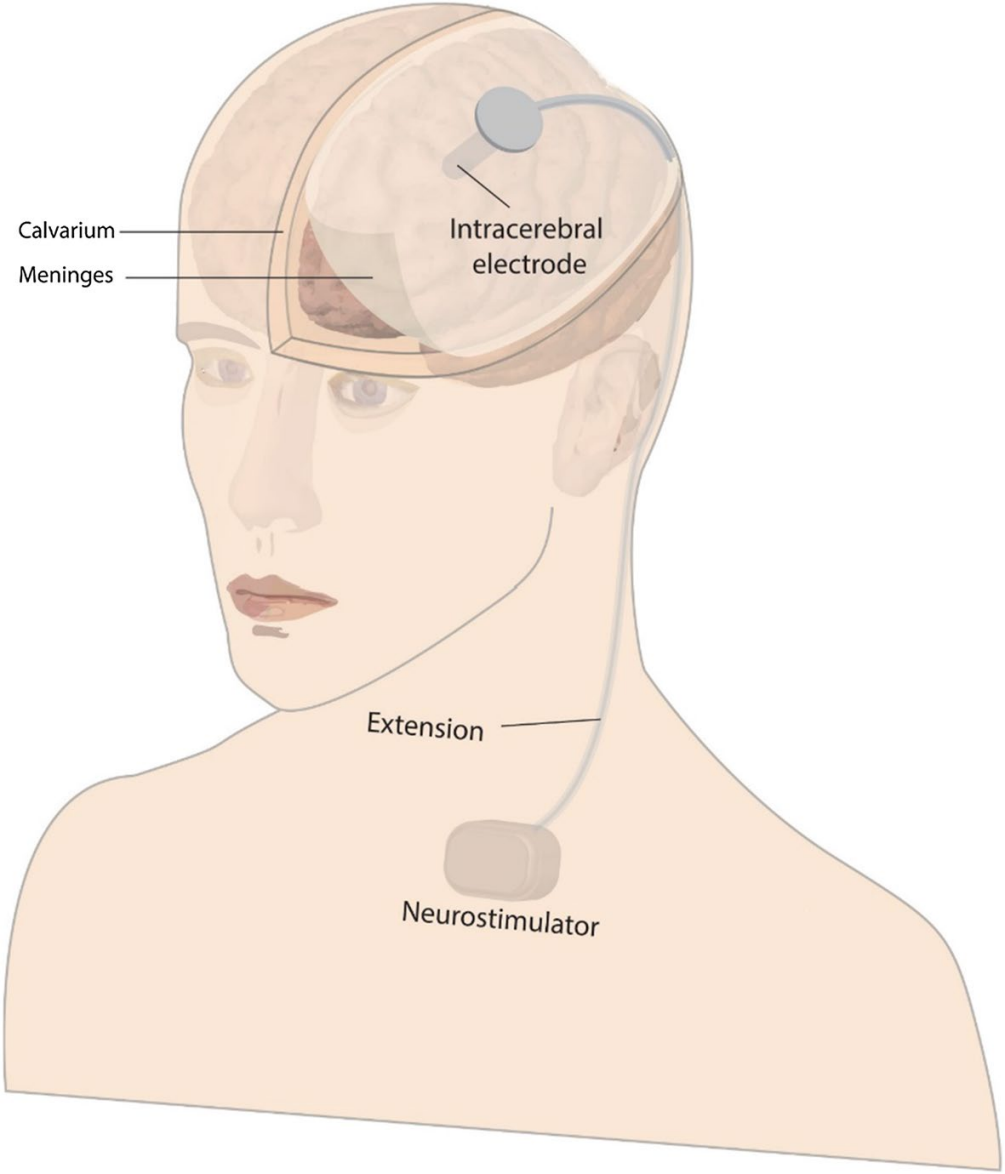
Electrode of deep brain stimulation is shown in this diagram

Electrodes have become necessary for activation as well as monitoring neurochemical levels, because deep brain stimulation is used to treat certain neurological disorders [301]. The triggered release of chemicals in the brain is thought to occur as a result of the stimulus. As a result, tracking the released chemical compounds emphasizes the significance of improving sensitive/responsive procedures to generate guidance as well as enhance the deep brain stimulation procedure's outcome [302]. Figure 3 depicts the placement of a 1.3-mm electrode with four 1.5-mm contacts in the brain for deep brain stimulation effects. Targeted stimulation of a particular and accurate site using a much thinner electrode, as well as feedback/guidance from calculated neurotransmitter rates, seems to be ideal [303]. Nanoelectrodes offer the opportunity to face this obstacle, with an extremely high susceptibility of about 1 nM, a quick velocity of 10 ms resolution, as well as long-term implant placement stability and reliability [304].

All of the aforementioned nanostructured materials have been employed to diagnose neurochemicals. Here is a selection of studies that have been reviewed. According to Baldrich et al., SWCNT electrodes were created by entrapping nanotubes onto the exterior of magnetic particulates coated with protein. At a 200 μM concentration level, this electrode could differentiate the peaks of uric acid (UA) and DA. Li et al. used SWCNTs to modify electrodes of glassy carbon (GCEs) to achieve a DA detection limit of 50 nM with a peak current that ranged in a linear fashion between 5 and 100 μM [305]. Moreno et al. were able to achieve a linear correlation throughout the region of 50 nm to 1 μM in the existence of AA using the MWCNTs-modified graphite electrode that was pretreated for DA detection [306]. Komathi et al. investigated electrodes for responsive DA sensing leveraging a nano-based composite of MWCNTs, gold nanostructures, and sol–gel silica [307]. Without any of the gold nanomaterials, the susceptibility was observed to be almost four times lower. MWCNTs, which are hydrophobic in nature, and the silica containing an –NH2 group, which is water-soluble, are both found throughout the porous structure of the silica matrix. The latter absorbs and accepts ascorbate ions, thus resisting DA molecules as they lie on the external surface of MWCNTs which undergo extreme electron transfer transformations or reactions. Furthermore, the DA electrocatalytic oxidation is facilitated by the existence of gold nanostructures. The linear range for DA recognition was determined to be 0.1 to 30 nM in the research. Yang et al. manufactured a nanoscaled composite of MWCNTs and copper oxide and Nafion [308]. They utilized it to modify or adjust a GCE with a limit of detection of 0.4 μM. To avoid nanotube aggregation, Aravind and Ramaprabhu established a composite of Pt nanostructures and MWCNTs [309]. Eventually, they employed SS-DNA to immobilize the prepared composite. The SS-DNA further facilitated the DA electron transfer reaction. The DA limit of detection for this composite electrode was found to be 0.8 μM. After administering a synthetic precursor of 5-HT into an anesthetized rat, Kumaraswamy and Venton altered and modified a microelectrode of carbon fiber using SWCNTs and exploited it to diagnose ST and DA in vivo inside the striatum [310]. The levels of 5-HT and DA were determined 24 min after infusion and were 130 nM for 5-HT and 250 nM for DA.

Kim et al. modified a glassy carbon electrode (GCE) with GR to decrease capacitive ambient flow and enhance DA current pressure [311]. As a result of the research, they were able to achieve a full peak distinction between AA and DA with a DA diagnosis linear range of 4 to 100 μM. Alwarappan et al. employed a GCE modified by GR as well, but only published findings for 1 μM of AA, 5-HT, and DA [312]. Sun et al. used graphene/Pt to modify a GCE to achieve distinguished peaks of DPV curves for DA, UA, and AA [313]. The GC/GR and GC electrodes were outperformed by self-assembled Pt particulates with a diameter of 1.7 nm on GR. Tan et al. functionalized GCE by applying a nano mixture structure of GR sheet and β-cyclodextrin to accomplish a continuous current reaction with DA thresholds ranging from 9 nM to 12.7 μM at PBS [314]. In the amperometric mode, the linear range was from 0.9 to 200 μM. In both cases, the GCE adjusted by nanocomposite outperformed the GC/GR and GC electrodes. It, therefore, was directly attributable to the mass transfer regulation, including its dopamine electrochemical reaction on the nanocomposite, rather than the normal mechanism of limited adsorption. For the recognition of AA, DA, and UA, Han et al. modulated an electrode of glassy carbon with a composite of chitosan and graphene [315]. Then, they compared the prepared composite to an electrode of GC–chitosan. The introduction of GR to increase the electrocatalytic activity was found to be beneficial for the oxidation reactions of all three substances. Gao et al. adjusted an electrode of glassy carbon using graphene oxide and discovered a detection threshold of 0.27 μM DA throughout the existence of AA, as well as a linear relationship between concentration level and the oxidation-related current peak of 1.0–15.0 μM [316]. In the existence of 1000-x levels of UA and AA. Thomas et al. used graphene oxide to modulate and modify a carbon paste electrode to accomplish a recognition threshold of 15 nM for DA [317]. Sun et al. generated a graphene–tin oxide nanosheet nanocomposite and utilized it to modulate a liquid electrode of carbon ions [318]. With a limit of detection of 0.13 μM, this modified/modulated electrode produced peak currents proportional to the concentration of DA in the range between 0.5 and 500 μM. Tsai et al. altered GCEs by coating them with Te nanowires and then covering the modified electrode with nafion to increase its selectivity and stability [319].

Chandrashekar et al. developed a biopolymer by electropolymerizing L-arginine upon the electrode of carbon paste and using it to distinguish AA, UA, and DA [320]. The intensity of the peak throughout the CV calculation revealed a linear response of 50 μM to 0.1 mM for dopamine concentration, with a limit of detection of 0.5 mM. Diamond electrodes have also been investigated in addition to GR and CNTs. Raina et al. developed steady flow curves of CV for DA concentrations ranging from 100 to 800 μM in 0.1 M PBS using a nanodiamond ultramicroelectrode array incorporated with nitrogen [321].

BioFETs have been utilized to track dopamine in addition to electrochemical methods like the ones described above. Li et al., for instance, manufactured an open gate field-effect transistor of sensitive ions; then assessed the I–V properties as DA levels increased from 1 fM to 1 nM [322]. Nevertheless, no information was provided about how the system will work once DA is contained in UA and/or AA mixtures.

## Neuroimaging

One of the most effective techniques for studying CNS diseases is the capacity to visualize the brain, which is a crucial milestone in gaining novel clues into improved therapeutic interventions on the basis of improved diagnostics [323]. Whole-brain screening, in particular, can record the functional and structural fluctuations of neural communications in the undamaged nervous system, allowing for a better grasp of the neural activity rhythms taking part in experience-dependent structural plasticity [324, 325]. Various neurodegenerative and psychiatric diseases need these findings to diagnose their clinical progression [326, 327]. Existing whole-brain imaging methods, on the other hand, have a number of limitations that hinder the volume of information that can be acquired, such as inadequacy of responsiveness to particular clinical diagnostic biomarkers, a short half-life following intravenous injection, and poor blood–brain barrier penetration. Furthermore, some whole-brain imaging methods only assess alternative indicators of brain function and may not represent actual brain activity [328].

Molecular imaging modalities, on the other hand, have been shown to be very useful in exploring the more precise mechanisms of neuronal activity induced by synaptic processes arising from special molecular interactions [329, 330]. In vivo, optical fluorescence microscopy has been remarkably advantageous in disclosing many of the various process steps of disease pathology at the neuronal resolution, and also assess the outcomes of investigational therapeutic approaches on special neuronal subpopulations, thanks to enhanced sophistication in all laboratory animals with neural disorders [331]. In addition, molecular imaging of neurons has become a key method for investigating the functionality of live organisms owing to its non-destructive character, high susceptibility, and the utilization of widely accessible and relatively affordable equipment as compared to other imaging techniques. However, there are still major challenges in investigating neural growth processes, including artefact interference and phototoxicity induced by fluorescent probe instability [332]. Due to the limitations of existing neuroimaging methods, innovative technical advancements have been developed that have significantly improved the state-of-the-art. Manufactured nanostructures with a variety of surface chemistries as well as excellent optical properties can be used to overcome the difficulties of in vivo neuroimaging methods. Because of their distinctive optical, chemical, and physical characteristics in the nanometer range, several nanomaterials have already been explored for application in biomedical image analysis during the past decade [333]. Innovative medical imaging techniques have sprung up as a result of contemporary breakthroughs in the synthesis, engineering, and functionalization of different nanostructures. These nanoscale probes are essential nanosystems for visualizing, characterizing, and quantifying biochemical mechanisms in live organisms at various imaging stages [334].

## Therapeutic strategies

**Fig. 4:**
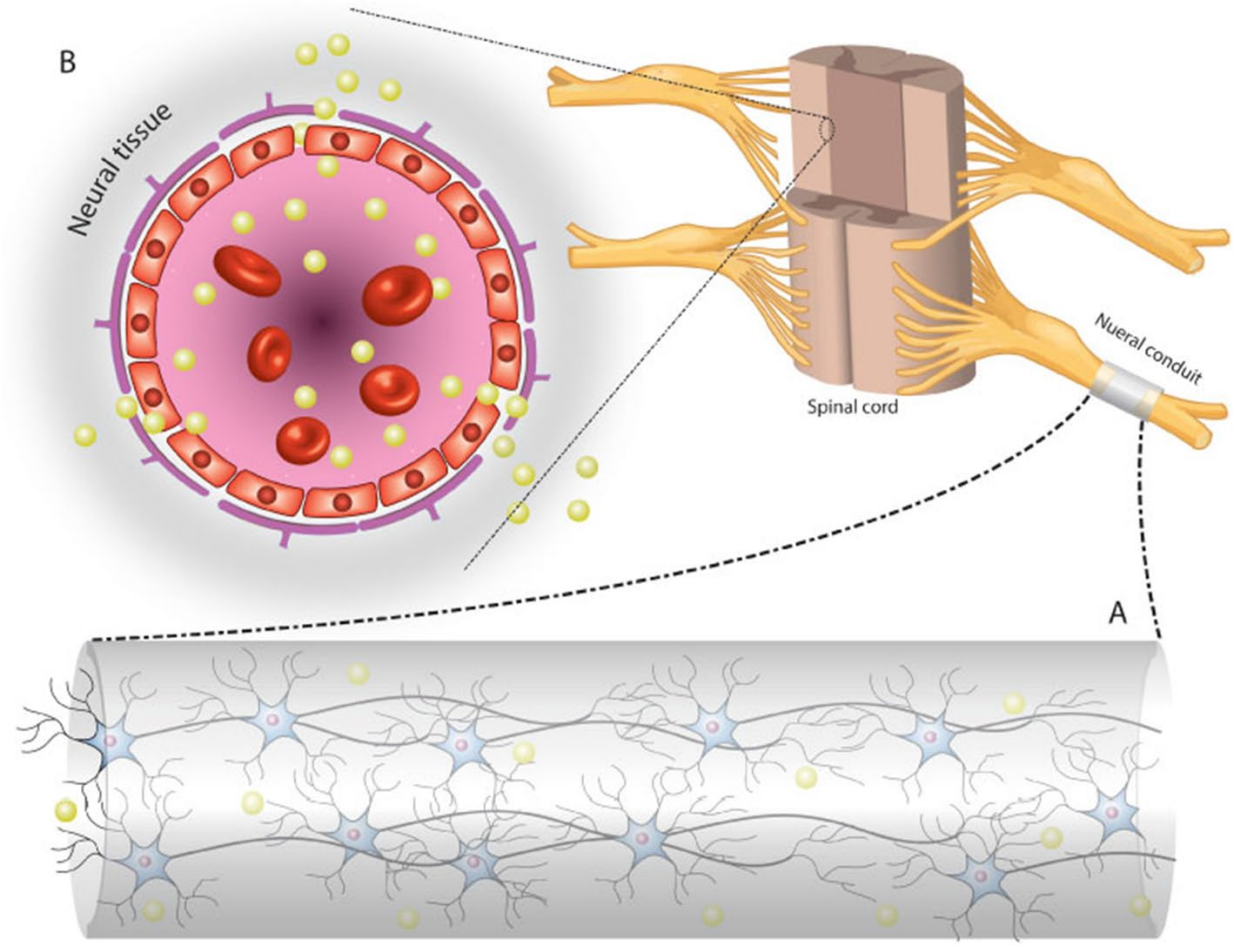
Advantages of nanotechnology in clinical neuroscience. Nanotechnology has the potential to restrict or reverse neuropathological disorder mechanisms at the molecular scale, as well as promote and assist other methods. **A** Nanoengineered scaffold (neural conduit) includes functional nanoparticle components that imitate the extracellular matrix to offer a physical and bioactive microenvironment for neural regeneration. **B** Techniques including focused ultrasound or osmotic (mannitol) shock have been used to temporarily open the BBB and allow nanoparticles to enter the cells to facilitate the transportation of medications through nanomaterials

**Fig. 5:**
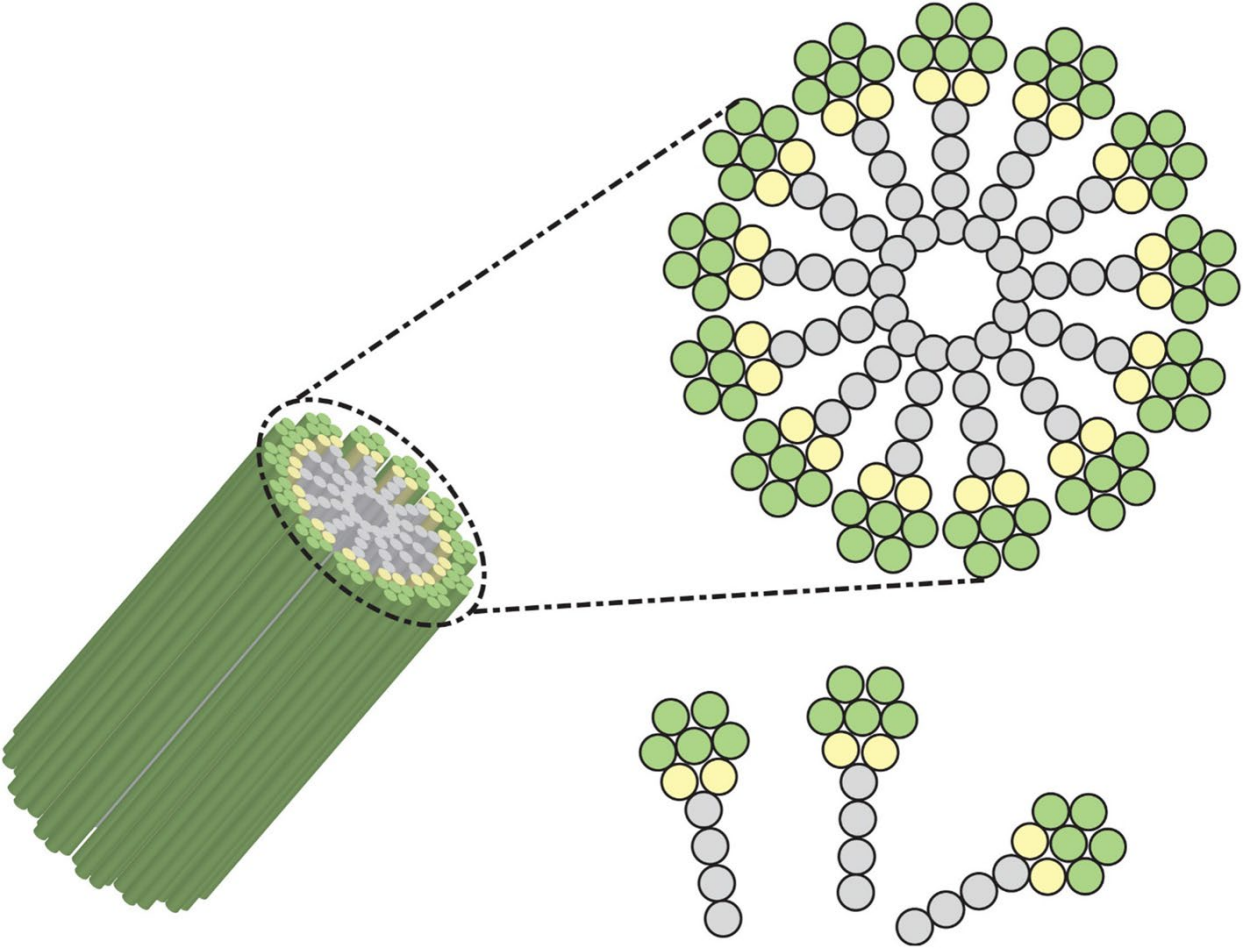
Nanomaterials engineered to very precise specifications result in the activation of targeted cellular interactions that may help to achieve certain neurological goals. One way to build up a thick nanofibre matrix is using peptide–amphiphile molecules, which contain a hydrophobic carbon tail (white circles) and a hydrophilic head group of peptides (green circles), linked by a peptide spacer area (yellow circles). Neural stem cells and progenitor cells can be enclosed under physiological circumstances, but they can self-assemble and create a gel containing neural progenitor cells and stem cells in which they are trapped. To do this, neural progenitor cells, as well as stem cells' development and differentiation, may be regulated in this manner

Nanotechnology applications are aimed at limiting and reversing neurological diseases by improving neural regeneration (Fig. 4) [36, 335]. Tissue engineering strategies based on bulk material manipulation are developing toward the fabrication of nanoengineered scaffolds which facilitate and enhance neurite and axonal development [336, 337]. Poly-(l-lactic) acid (PLLA) as well as other manufactured hydrogels with tailored microscale characteristics, and also scaffolds generated from naturally available substances, such as collagen, are instances of tissue engineering at the micron-scale [338]. PLLA scaffolds with an ultrastructure comprised of formed PLLA fibers with dimensions of 50–350 nm and porosity of 20–85% are one instance of a nanoengineered system developed from this kind of research. Rather than casting the scaffolds on glass, they were made employing liquid–liquid phase separation through dissolving PLLA in tetrahydrofuran (THF). Neonatal mouse cerebellar progenitor cells were capable of extending neurites and differentiating into mature neurons while growing on the scaffolds. The nanofibre network self-assembly comprised of peptide-amphiphile molecules is a radically new strategy for the manufacture of nanostructured materials that encourages and promotes neural regeneration (Fig. 5) [339]. When molecules with a hydrophilic peptide head group and a tail containing hydrophobic carbon are introduced to physiological ionic environments, they are self-assembled into a nanofibre dense network. At the macroscopic scale, it captures the neighboring water molecules, resulting in the formation of a poor, self-supporting gel. Ile‐Lys‐Val‐ala‐Val (IKVAV), a bioactive peptide derived from laminin, which stimulates neurite branching and development, was used to create the head groups of hydrophilic peptides that constituted the exterior of the fibers [340–342]. In nanofibre networks, neural progenitor cells encapsulated from the cortex of an embryonic mouse led to rapid and persistent neuronal differentiation (respectively, at 1 and 7 days, 30% and 50% of neural progenitor cells differentiated into neurons in vitro), with little astrocytic differentiation (respectively, at 1 and 7 days, 1% and 5% of neural progenitor cells differentiated into astrocytes in vitro). As a result, this method could enhance neuronal differentiation in an injured region while also reducing the consequences of glial scarring and reactive gliosis, two common neuropathological disease mechanisms.

Free radicals are toxic, and they increase the risk of many diseases. Nanotechnology has been used to mitigate this threat by decreasing the negative impact of free radicals that result from injury, which is a significant neuropathological mechanism that causes neurotrauma, ischemia, and degenerative diseases [343, 344]. Fullerenols have been shown to exhibit antioxidant properties, which are due to the presence of hydroxyl groups on functionalized fullerene structures (molecules made up of periodic carbon atom arrangements) [345]. They additionally act as free radical scavengers, which may reduce NMDA (*N*-methyl-d-aspartate)-, AMPA-, glutamate-, and kainate-induced apoptosis and excitotoxicity [346–349]. Neuroprotection facilitated by fullerenol has been demonstrated in vitro and in vivo [350]. Fullerenol was shown to have protective effects in an animal model of familial amyotrophic lateral sclerosis. In cultured neuronal cells, it reduces apoptosis and excitotoxicity while slowing the onset of motor degeneration in vivo [351, 352]. Because fullerenols showed no impact on GABA_A_ or taurine receptors, their neuroprotective action may be mainly mediated through the blockage of glutamate receptors. They effectively reduced glutamate-induced intracellular calcium increases, which is a key process in neuronal excitotoxicity.

The construction of functionalized nanostructures that can be delivered systematically and transport medicines as well as small chemicals over the blood–brain barrier is another therapeutically important area of intensive study [353, 354]. For the therapeutic intervention of a broad variety of neurological diseases, this is a key clinical goal. Different substances and chemically synthesized strategies are being researched to accomplish this. Crosslinked polyethylenimcine and poly(ethylene glycol) gels have been used to transport oligonucleotides [355]. Charge imbalances in the electrostatic forces between the oligonucleotides that are spontaneously negatively charged and the gels offer a reversible delivery system for transporting chemicals over the blood–brain barrier and subsequently releasing them. The NMDA receptor antagonist MRZ 2/576, neuropeptides (such as enkephalins), and chemotherapeutic agent doxorubicin, were absorbed on the surface of poly (butylcyanoacrylate) nanostructures covered with polysorbate 80 [264, 356–359]. The polysorbate on the nanomaterials' exterior binds to apolipoprotein E and B throughout the bloodstream, and the nanomaterials are picked up through capillary endothelial cells of the brain by receptor-mediated endocytosis [360]. The significant mortality rates related to common malignant neoplasms in the physically restricted regions of the spinal canal and cranium may allow nanomaterials to target tumors in the CNS as an especially applicable use of this technology.

Many strokes are addressed via restorative approaches that focus on cerebral parenchymal, endothelial, and cell health [361]. To have a functional CNS, communication between cells and signalling within the neurovascular unit are essential, especially at the multicellular brain–vessel–blood interface with its extremely selective blood–brain barrier. Crosstalk between cells is also mediated by exosomes, according to Zagrean et al. [362]. Additional study findings show that the restorative therapeutic efficacy of exosomes is apparent in patients with ischemic stroke, a common neurological disease that is still in need of a viable treatment. At this point, Pulgar discussed transcytosis across the blood–brain barrier [148]. Therefore, our attention is drawn to "receptor-mediated transcytosis" (RMT) by Pulgar, which operates inside the brain endothelial cells to transport cargo to the brain parenchyma [148]. These advancements in RMT-mediated brain medication delivery are very important.

## Present challenges and offer opportunity

With regard to neuroscience, one of the greatest challenges that nanotechnology presents is how complex it is. We acknowledge that this information has a major effect on our ability to intervene at the molecular level, as well as how the nervous system operates, malfunctions in disease, and how we might comprehend it. Neural cells may now be both positively and negatively influenced by molecules, giving rise to both favourable and unfavourable characteristics. The possibility of adapting technology to specific applications exists via the capacity to utilize medicines, small compounds, neurotransmitters, and brain developmental variables [4, 363]. Laminins, cadherins, and morphometric protein families of the bone, as well as their receptors, can be modified in directions we have not thought of before. Functional specificity is a benefit of integrating molecules into engineered materials and devices [364].

Laminins, which are made up of 12 different types of trimeric proteins with alpha, beta, and gamma chains, are an example of a trimeric protein with alpha, beta, and gamma chains [365–367]. There are many bioactive peptide sequences among the isoforms, and some of them have a distinct affinity for particular types of cells, resulting in diverse bioactive responses. For instance, at least 48 different short peptide sequences are present in the laminin 1 isoform, and several of these peptides facilitate neurite outgrowth and neuronal adhesion in distinct populations of neurons. These peptides (25 of 48 checked) help in promoting neurite outgrowth as well as neuronal adhesion in distinct populations of neurons. There are many signaling molecules in the nervous system that have an effect on growth and behavior [342]. Because of this, these molecules, as well as laminins, may be used to manufacture extremely selective nanotechnologies. This technique enables any desirable cellular signalling route to be targeted.

As shown above, one of the major difficulties that nanotechnology faces in neuroscience is the necessity for increased specificity. In addition, numerous induced physiological activities, with minimum adverse consequences, are necessary [36, 368]. Target cell and tissue interconnections are essential to increase the magnitude and appropriateness of the physiological effects as well as to minimize side effects. A critical issue is the need for systems that are capable of multitasking, such as targeting several receptors or ligands simultaneously. Successful attempts to treat multi-dimensional CNS diseases that arise from many interdependent molecular and biochemical processes are aided using an interdisciplinary approach. This is essential in addressing complex CNS diseases, such as the many interdependent molecular and metabolic processes that are the cause of many of these conditions. Severe brain damage or spinal cord damage may sometimes lead to subsequent harm. Despite the current capabilities of this kind of nanotechnology, however, these criteria have not yet been fully met to interface with the nervous system.

The most noteworthy aspects of nanotechnology's contributions to neuroscience will be those that have a deep understanding of the underlying biology and use this knowledge in the pursuit of new (and maybe yet-to-be-discovered) molecular details [21, 369]. When it comes to in vivo applications, the use of nanotechnology in the nervous system is difficult. One of the most distinctive aspects of the brain is its intrinsic complexity, including its tough and restricted nature. The sophisticated information processing that takes place in the nervous system is rooted in the presence of multi-dimensional levels of cellular interactions and the heterogeneity of cells (for instance, the spatiotemporal summation of postsynaptic potentials). It is necessary to be aware of the complexity of the CNS while using nanotechnologies that aim to influence it. Unpredicted and undesirable "side effects" throughout the various physiological systems or nervous systems may occur if this step is not implemented. In vivo applications of nanotechnology present a challenge, because they are not advanced for interacting with neurons at the subcellular and cellular levels but rather to target widespread systemic functional interactions, which typically require the coordinated efforts of many interconnected neurons and glia. So far, this kind of application has only been used in a small number of settings. Nevertheless, although technically and theoretically complex, these applications may play a large role in the advancement of clinical neuroscience. While much of this research is valuable, much of it is still left to be done.

In the discussion above, we refer to the use of nanotechnology in all areas of neuroscience [21, 369]. When it comes to in vivo applications, the use of nanotechnology in the nervous system is difficult. One of the most distinctive aspects of the brain is its intrinsic complexity, including its tough and restricted nature. The sophisticated information processing that takes place in the nervous system is rooted in the presence of multi-dimensional levels of cellular interactions and the heterogeneity of cells (for instance, spatiotemporal summation of postsynaptic potentials). It is necessary to be aware of the complexity of the CNS while using nanotechnologies that aim to influence it. Unpredicted and undesirable ‘side effects' throughout the various physiological systems or nervous systems may occur if this step is not implemented. In vivo applications of nanotechnology present a challenge, because they are not advanced for interacting with neurons at the subcellular and cellular levels, but rather to target widespread systemic functional interactions, which typically require the coordinated efforts of many interconnected neurons and glia. So far, this kind of application has only been used in a small number of settings. Nevertheless, although technically and theoretically complex, these applications may play a large role in the advancement of clinical neuroscience. While much of this research is valuable, much of it is still left to be done.

Although physically challenging, the nervous system is the second major factor to take into account when looking at possible in vivo applications of nanotechnology. Due to the vulnerability of the CNS to damage and harm's diminished possibility of permeating the blood–brain barrier and blood–retina barrier, the CNS is effectively shielded from both physical and mechanical trauma. The deployment of nanotechnologies in vivo must be carried out while minimally interfering with these structures in order for them to achieve their main purpose. This will be difficult to do. Local and systemic and adverse reactions related to the delivery and main purpose of the applied technology must be carefully studied and avoided, as with all things nanotechnological, to prevent unwanted outcomes. An active and significant investigation into the safety of nanomedicine is now being conducted. Even if all these difficulties are overcome, the promise of nanotechnology for both ex vivo and in vivo research and application offers huge potential for improving knowledge of normal physiology and creating therapeutic applications [370, 371]

## Conclusion

Nanoneuroscience is the merging of nanotechnology with what is understood regarding the nervous system, two fields that are developing quickly. The combination of these two fields may lead to a treatment for a variety of CNS diseases, including neurodevelopmental, motor, and sensory difficulties. This review describes the present state of nanotechnology in neural tissues. Neuroscience is exploring new methods utilising nanoscience, such as nanotools with innovative biomimetic designs, to create improved interfaces for the nervous system. This means that neuroscience and nanotechnology have a significant number of innovative strategies to investigate brain activity at their disposal, thanks to the simultaneous measuring and manipulating of the activity of thousands or even millions of neurons.

## Future directions

Researchers have already seen significant effects from applying nanotechnology to neuroscience, and these effects are expected to continue in the near future. Ex vivo and in vitro investigations of neural cells have benefited from short-term advancement, which frequently supports or augments conventional methods. These breakthroughs help us better comprehend cellular neurobiology and neurophysiology, as well as our knowledge and understanding of neuropathology.

Although nanotechnologies intended to interface with the nervous system in vivo are still in the early stages of research, they will have major therapeutic effects. Nanotechnologies aimed at assisting cellular or pharmacological treatments, as well as enabling direct physiological impacts in vivo, will have a substantial impact on clinical prevention and care. One of the key factors in the extensive potential of nanoscale technologies is the capacity to accurately interact/communicate with cells at the molecular level.

## Data Availability

All the data and materials supporting the conclusions were included in the main paper.

## Abbreviations

CNS: Central nervous system
FRET: Fluorescence resonance energy transfer
CNTs: Carbon nanotubes
GR: Graphene
AuNPs: Gold nanoparticles
SWCNTs: Single-walled carbon nanotubes
MWCNTs: Multiwalled carbon nanotubes
CVD: Chemical vapor deposition
GABA: Glutamate and γ-aminobutyric acid
NO: Nitric oxide
PIN: Protein inhibitor of neuronal NO synthase
PECVD: Plasma-enhanced chemical vapor deposition
CNFs: Carbon nanofiber-based
BioFETs: Bio-field-effect transistors
NE: Nor-epinephrine
EP: Epinephrine
ACh: Glutamate acetylcholine
5-HT: 5-Hydroxytryptamine
DSPE–PEG2000: Distearoyl-phosphatidyle–thanolamine–polyethylene glycol 2000
CHOL: Cholesterol
DSPC: L-α-distearoyl-phosphatidylcholine
PACA: Poly(alkyl cyanoacrylate)
PLGA: Poly(lactic-co-glycolic acid)
FDA: Food and Drug Administration
PLA: Poly(lactic acid)
PGA: Poly(glycolic acid)
PAMAM: Polyamidoamine
MPS: Mononuclear phagocyte system
RES: Reticulo-endothelial system
SLNs: Solid–lipid nanoparticles
NLCs: Nanostructured lipid carriers
ALS: Amyotrophic lateral sclerosis
DA: Dopamine
TBI: Traumatic brain injury
AA: Ascorbic acid
UA: Uric acid
GCEs: Electrodes of glassy carbon
GCE: Glassy carbon electrode
PLLA: Poly-(l-lactic) acid
THF: Tetrahydrofuran
NMDA: *N*-Methyl-d-aspartate
RMT: Receptor-mediated transcytosis
AMPA: Alpha-Amino-3-Hydroxy-5-Methyl-4-Isoxazole Propionic Acid
